# Nutritional Potential of Edible Insects as Alternative Ingredients in Fish Feed: A Path to Modern Aquaculture

**DOI:** 10.1155/anu/7009004

**Published:** 2025-10-28

**Authors:** Pietro Ragozzino-Paulino, Maria Lucia Cocato, Jorge Eduardo de Souza Sarkis

**Affiliations:** Nuclear and Energy Research Institute (IPEN), Lasers and Applications Center, Av. Prof. Lineu Prestes, 2242, São Paulo 05508–000, Brazil

**Keywords:** *Acheta domesticus*, aquaculture, *Bombyx mori*, edible insects, fish nutrition, *Hermetia illucens*, *Musca domestica*, *Tenebrio molitor*, *Zophobas morio*

## Abstract

The incorporation of edible insects into fish diets has gained increasing attention due to their rich nutritional profile and potential as alternative ingredients in aquafeeds. This review examines the composition of key insect species, including *Hermetia illucens*, *Tenebrio molitor*, *Musca domestica*, *Zophobas morio*, and *Bombyx mori*, comparing their macronutrient and micronutrient profiles, as well as their applicability in dietary formulations for fish. Studies indicate that these insect meals can effectively replace fishmeal (FM) in varying proportions without compromising growth performance, nutrient digestibility, immunological response, or feed efficiency in both freshwater and marine species. Furthermore, their amino acid and fatty acid profiles closely align with the nutritional needs of farmed fish, supporting optimal health and development.

## 1. Introduction

It is projected that by 2050, agricultural production will need to increase by 50% to meet the demands of a global population that is expected to reach 9.7 billion people [1–3]. This significant population growth, along with an anticipated rise in living standards in developing countries, will lead to a marked increase in the consumption of animal-based foods. As a result the search for sustainable alternative protein sources is imperative [4, 5]. In this scenario, aquaculture stands out as a vital strategy for ensuring global food security, ranking among the fastest-growing food sectors worldwide. Moreover, it provides a robust alternative to capture fisheries, which face increasing challenges from overfishing and the degradation of aquatic ecosystems [6–9].

Feed is one of the main limiting factors in aquaculture. Among the various ingredients used, fishmeal (FM) and fish oil (FO) are highly valued due to their balanced amino acid profile, high digestibility, and presence of long-chain polyunsaturated fatty acids (LC-PUFAs) [10, 11]. However, the increasing demand for these ingredients has placed significant pressure on natural fish stocks, affecting their availability and costs [12, 13]. Between 2000 and 2017, the annual catch of forage fish used for FM and FO production declined from 23 to 16 million tons, which led to a decrease in global FM production from 6.6 to 4.8 million tons [7, 14, 15]. Similarly, FO production saw a significant drop, declining from approximately 1.5–1.0 million tons annually [16–18]. As a result, the prices of these ingredients have continuously increased since the 2000s, leading to a gradual reduction in their use in aquafeed formulations [19].

The quest for nutritionally adequate alternatives that fulfill the physiological and metabolic requirements of fish is critical in today's aquaculture [20–23]. Plant-based ingredients such as grains (wheat and corn), oilseeds (soybean, rapeseed, and cottonseed), and pulses (beans, lupins, and peas) are widely available and cost-effective, but they may contain antinutritional factors that limit their digestibility and use in certain species [24–35]. However, while their use helps alleviate pressure on natural fish stocks, it simultaneously increases demand on agricultural production for feed. By-products from poultry, ruminants, and swine represent another promising alternative, offering rich sources of proteins and essential amino acids. Nonetheless, regulatory challenges and the need for long-chain fatty acids supplementation may hinder their acceptance [36–41]. Despite the extensive research highlighting their potential for aquafeed formulation, these sources still face significant nutritionally and sustainability hurdles.

In this scenario, the use of edible insects stands out as a highly promising alternative. Their economic and environmental advantages can substantially enhance the efficiency and feasibility in aquafeed formulation [42–48], positioning them as a viable solution for future aquaculture needs. Embracing this innovative approach could lead to more sustainable practices in the industry, ensuring the health of fish populations while meeting the demands of a growing global market.

### 1.1. Edible Insects

Insects represent the largest class within the phylum Arthropoda and have been used as a food source for humans throughout history [49]. Over the past two decades, interest in their potential as an alternative source of high-quality protein has grown significantly. In addition to being rich in proteins, lipids, essential amino acids, and minerals, insects exhibit high bioconversion efficiency and can be reared at high densities [50–52]. Their ability to feed on decomposing organic matter makes them particularly suitable for circular supply chains, where they can recycle waste from the agro-food industry into valuable protein for animal feeds [53]. Some species have been identified as key organisms in converting organic waste into high-value protein and fat, reinforcing their role in circular bioeconomy models [54–57]. Additionally, insects are cold-blooded, which results in highly efficient food conversion compared to conventional livestock. They also generate lower greenhouse gas emissions and ammonia levels, contributing to a reduced environmental impact [58, 59]. By utilizing food waste that would otherwise be discarded or incinerated, insect farming further enhances sustainability, providing a promising alternative to traditional livestock production [60, 61]. The following sections discuss six key insect species (*Hermetia illucens*, *Musca domestica*, *Zophobas morio*, *Tenebrio molitor*, *Acheta domesticus*, and *Bombyx mori*), highlighting their nutritional composition as presented in Table 1.

### 1.2. Black Soldier Fly (*Hermetia illucens*)

The black soldier fly (*Hermetia illucens*; Diptera: Stratiomyidae) has been extensively investigated as a promising alternative to FM in aquaculture due to its high nutritional value and environmental sustainability [111]. Its larvae stand out for their ability to feed on a wide variety of organic waste, reducing its volume and contributing to the circular economy [112–115]. Furthermore, its well-balanced nutritional composition, rich in proteins, lipids, essential amino acids, and vitamins (Tables 1, 2 and 3), makes *H. illucens* meal a viable feed source for animals [167, 168].

With a short life cycle of approximately 3 weeks and ease of cultivation, this species is highly efficient for large-scale production [169, 170]. During the prepupal stage, larval harvesting is simplified, reducing operational costs and making production economically attractive [169, 171]. *H. illucens* is widely distributed in temperate, subtropical, and tropical regions, thriving optimally between temperatures of 25°C and 30°C. It has a low tolerance for cold and cannot survive at temperatures below 5°C [117, 148, 172]. The life cycle of *H. illucens* undergoes five stages: egg, larva, prepupa, pupa, and adult. The larval and pupal phases are particularly nutrient-rich, with their composition varying based on the provided diet [173–175]. In addition to its use in fish feed, *H. illucens* meal has demonstrated antimicrobial properties, helping control bacteria such as *Salmonella* and *Escherichia coli*, reinforcing its safety as a feed ingredient [176, 177]. These characteristics make *H. illucens* a sustainable and highly versatile alternative for aquaculture and other animal feed industries [122, 178, 179].

### 1.3. Common Housefly (*Musca domestica*)

The housefly (*Musca domestica L*.) is a globally distributed species predominant in various regions [180]. Its larvae are highly nutritious, possessing a well-balanced amino acid profile and high levels of proteins and lipids (Tables 1 and 2), making them a viable alternative to FM in aquaculture [135]. *M. domestica* larvae meal contains significant concentrations of lysine, threonine, and methionine, making it an ideal supplement for cereal- and legume-based diets that are protein-deficient. This improves fish growth while providing essential energy, proteins, and micronutrients [181].

Additionally, these larvae contain biologically active compounds such as antimicrobial proteins, lectins, and chitin, which have applications in industry, medicine, and agriculture [178, 180]. One of the primary advantages of *M. domestica* cultivation is its high reproduction rate and ability to bioconvert organic waste, including food scraps and cattle manure, significantly reducing waste volume and contributing to the circular economy [178, 182]. However, the efficiency of this process can be influenced by environmental factors such as temperature variations, increased pH to alkaline levels, ammonia release, and moisture reduction, which may affect larval development and waste conversion efficiency [178]. Due to its high nutritional value, low production cost, and simplified processing, *M. domestica* larvae meal presents great potential for application in aquaculture and animal feed in a sustainable manner [183, 184].

### 1.4. Superworm (*Zophobas morio*)

The superworm (*Zophobas morio*; Coleoptera: Tenebrionidae) has gained increasing interest as a viable alternative for animal feed and aquaculture due to its high nutritional value and broad availability [80, 185]. Native to tropical regions of Central and South America [186–188], *Z. morio* is widely farmed for feeding birds, reptiles, and fish and is also consumed by some human populations in Mexico [189]. In Brazil, it is exclusively produced for animal nutrition [153].

The larvae of *Z. morio* exhibit a high protein content, reaching up to 49 g/100 g of dry matter, and a lipid content of up to 42 g/100 g (Table 1). Its fatty acid profile comprises 44%–47% saturated fatty acids (SFAs), 31%–32% monounsaturated fatty acids (MUFAs), and 22%–24% polyunsaturated fatty acids (PUFAs) [80, 155, 185] (Table 4). Additionally, *Z. morio* contains significant levels of essential amino acids, chitin, and bioactive compounds, some of which have antimicrobial and prebiotic properties beneficial to animal health [154, 214–216]. The farming of *Z. morio* is similar to that of *Tenebrio molitor*, characterized by a high reproductive rate, with females laying over 2000 eggs throughout their life cycle [185]. However, reliable scientific data on large-scale production and its incorporation into the diets of different animal species remain limited. The increasing research interest in *Z. morio*, including the complete sequencing of its mitochondrial genome [217], highlights its potential as a promising ingredient for sustainable animal feed [54, 218, 219].

### 1.5. Mealworm (*Tenebrio molitor*)

The mealworm (*Tenebrio molitor*; Coleoptera: Tenebrionidae) has been extensively studied as an alternative protein source for aquaculture and livestock due to its high nutritional value and large-scale production feasibility [87, 215, 220, 221]. Its larvae contain protein levels ranging from 47% to 63% and lipid content between 31% and 41% (Table 1), with an amino acid profile compatible with the nutritional requirements of aquatic animals [205]. Additionally, *T. molitor* larvae are rich in unsaturated fatty acids (Table 4), serving as a beneficial lipid source for livestock, and contain bioactive peptides that promote animal health [222–227].

Recent studies indicate that *T. molitor* meal can replace 33% to 74% of FM and has been evaluated as a viable alternative in poultry and cattle feed [42, 135, 228, 229]. The acceptance of fresh and dried *T. molitor* larvae as an ingredient for aquaculture has been widely validated in nutritional trials, further driving its replacement of traditional protein sources to meet the growing demand for sustainable aquafeed solutions [184, 230].

One of the key advantages of *T. molitor* is its ability to thrive on low-nutritional-value substrates, such as agro-industrial by-products, combined with high feed conversion efficiency, lower water and space requirements, and the potential to reduce greenhouse gas emissions [52, 231, 232]. However, several factors can influence its nutritional value, including diet, genetic selection, environmental conditions, and postharvest processing, requiring strict control to optimize larval biomass quality [205]. Due to these characteristics, *T. molitor* represents a nutritionally rich and environmentally sustainable alternative for future animal feed formulations [87, 132, 199].

### 1.6. House Cricket (*Acheta domesticus*)

The house cricket (*Acheta domesticus*; Orthoptera: Gryllidae) has been extensively studied as a sustainable and nutritious ingredient for animal feed. Native to tropical regions where temperatures favor rapid growth, this species can be found in various habitats worldwide, except in cold climates above 55° latitude [233]. *A. domesticus* has a crude protein content ranging from 55% to 73% (Table 1) and contains significant proportions of MUFA and PUFAs (Table 4) [234, 235]. Its amino acid profile is well-balanced (Table 2), although it may be deficient in lysine and methionine, which can be supplemented in fish diets [129, 198].

Beyond its high protein content, *A. domesticus* is an excellent source of vitamins, including riboflavin (B2), pantothenic acid (B5), biotin (B7), and folate (B9), as well as essential minerals such as calcium (Ca), potassium (K), magnesium (Mg), phosphorus (P), sodium (Na), iron (Fe), zinc (Zn), manganese (Mn), and copper (Cu) [129, 154, 161, 198, 236] (Table 3). Recent studies also highlight its chitin content (~8.7%), which, when included in fish diets, enhances the interaction between chitosan-derived glucosamine and bacterial cell walls, reducing microbial populations and potentially benefiting gut health [237, 238]. However, the nutritional composition of *A. domesticus* can vary depending on developmental stage, diet, and processing methods [239]. Nevertheless, its nutritional versatility and high reproductive rate establish it as a sustainable alternative to replace conventional ingredients in aquafeeds [94, 216, 240, 241].

### 1.7. Silkworm (*Bombyx mori*)

The silkworm (*Bombyx mori*; Lepidoptera: Bombycidae) is widely cultivated for silk production and is one of the most important insect species used for both animal and human consumption. Its domestication dates back over 4000 years in China, and today, it is a valuable byproduct of sericulture, with approximately 8 kg of pupae generated for every 1 kg of silk produced [242]. While various *Bombyx* species can be commercially exploited, *B. mori* is the most domesticated and commonly farmed due to its exclusive diet of mulberry leaves [101, 102, 243].

Silkworm pupae represent the most nutritionally dense stage and are frequently processed into *B. mori* meal, a protein- and amino acid-rich ingredient [139]. The nutritional composition of *B. mori* is remarkable, with a protein content of approximately 56% (dry matter basis) and a lipid content of around 32.2% (Table 1) [103]. Additionally, silkworm pupae contain 17 of the 20 common amino acids (Table 2), including all nine essential amino acids, making them an excellent protein source for aquafeeds. Lipids extracted from silkworm pupae are rich in unsaturated fatty acids, such as linoleic acid, α-linolenic acid, and the omega-3 fatty acids eicosapentaenoic acid (EPA) and docosahexaenoic acid (DHA) (Table 4), which are crucial for fish nutrition [139, 140, 244]. Given its well-balanced amino acid profile, beneficial lipid content, and its role in the circular economy of sericulture, *B. mori* emerges as a highly nutritious and sustainable ingredient for aquafeeds.

### 1.8. Insect Composition Versus Fish Requirements

The consumption of insects is also well-documented as a natural part of the diet of various fish species, particularly during the early life stages of carnivorous and omnivorous fish, as well as in freshwater and marine species that tolerate different salinity levels [245–251].

Given this context, large-scale insect production as an alternative protein source for aquafeeds has expanded significantly in China, Europe, North America, Australia, and Southeast Asian countries, reflecting the growing demand for sustainable solutions in aquaculture [42, 229, 252]. However, the suitability of a given insect species for fish nutrition depends on its macronutrient and micronutrient profile, which can vary according to life stage, rearing conditions, and diet. These factors are particularly relevant when comparing insect composition with the specific nutritional requirements of different aquaculture species [42, 150].

### 1.9. Proteins and Amino Acids

The protein supply in the diet is one of the main factors influencing the productivity of farmed fish, as it is essential for tissue building and maintenance, as well as for the synthesis of enzymes, hormones, and other metabolites [253, 254]. From a zootechnical perspective, it is crucial that diets contain adequate protein levels to meet the needs of growing, maintaining, and repairing tissues, ensuring an optimal physiological state. In fish, protein typically represents the primary dietary component in terms of proportion [255]. The protein requirements for maximum growth have been extensively studied in juveniles of various aquaculture species, ranging from 40% to 50% crude protein, with even higher requirements in larvae and fry, especially in carnivorous species [254].

According to the literature, the crude protein content in insects can vary considerably, as indicated in Table 1, influenced by factors such as developmental stage, diet, and processing methods [133, 256, 257]. *Bombyx mori* exhibits one of the highest reported protein levels, ranging from 21.5% to 80.0%, while *Tenebrio molitor*, *Zophobas morio*, *Acheta domesticus*, and *Musca domestica* show broad variability, with values between 8.8% and 76.2% [72, 81, 82, 88, 104]. Conversely, *Hermetia illucens* has a more stable protein composition, ranging from 29.9% to 56.1%, making it one of the most commonly used species in feed formulations due to its high feed conversion efficiency and viability for large-scale production [62, 63, 258]. The protein content of these insect species makes them comparable to conventional sources such as soybean meal (~50% protein) and high-quality FM (up to 73% protein), demonstrating their potential as partial replacements for these raw materials in aquaculture diets [42, 73, 81, 198, 259].

Although the amount of protein is a key factor in aquafeed formulation, its quality is directly related to the essential amino acid profile [23]. In general, the amino acid composition of insects varies according to taxonomic order. Insects of the order Diptera exhibit profiles more similar to FM, while those of the orders Coleoptera and Orthoptera have profiles closer to soybean meal, potentially showing deficiencies in lysine or methionine [198]. Most studied insect species contain adequate levels of essential amino acids to meet fish nutritional requirements (Table 5), and in some cases, these values even exceed recommendations [129, 260]. *H. illucens*, for instance, has high levels of valine (2.29–6.79 g/100 g), leucine (2.67–7.83 g/100 g), and lysine (2.30–7.40 g/100 g), making it a strong candidate to replace FM due to its particularly high lysine content [62, 116]. *M. domestica*, on the other hand, contains elevated levels of phenylalanine (1.56–10.20 g/100 g) and threonine (1.35–7.60 g/100 g), while *T. molitor* and *Z. morio* stand out for their high leucine (up to 10.60 and 7.68 g/100 g, respectively) and arginine (up to 7.10 and 8.53 g/100 g, respectively) contents [81, 88, 129, 184].

However, some limitations must be considered. The low levels of essential sulfur-containing amino acids, such as methionine and cysteine, in certain insect species may indicate the need for supplementation or combination with other protein sources to ensure a balanced nutritional profile. This is particularly evident in *A. domesticus* (0.30–1.40 g/100 g) and *Z. morio* (0.21–2.02 g/100 g) [42, 261]. Additionally, tryptophan levels are notably low in most analyzed species, ranging from 0.07–1.50 g/100 g in *B. mori* to 0.15–1.00 g/100 g in *T. molitor*, suggesting that dietary formulations may require adjustments to meet the specific nutritional needs of different fish species.

### 1.10. Lipids and Fatty Acids

Lipids play a fundamental role in fish nutrition by providing essential fatty acids, improving meat texture, stimulating growth, and supporting reproduction. Additionally, they supply at least twice as much energy per gram as proteins or carbohydrates [262–264]. The energy requirements of fish vary depending on their size, species, feeding habits, and environmental conditions. In general, diets containing lipids between 10% and 20% promote optimal growth rates without leading to excessive fat accumulation in the carcass [265]. Compared to mammals, fish have relatively low energy demands due to their poikilothermic nature and lower energy expenditure for buoyancy regulation [266, 267] and ammonia excretion [268]. Studies indicate that both lipid deficiency and excess can impair fish growth through two distinct mechanisms: nutritional deficiency and metabolic dysregulation [269–271]. Therefore, maintaining a balanced lipid composition in formulated diets is essential to optimizing zootechnical performance while preventing negative physiological effects.

Insects tend to accumulate lipids during the larval stages, leading to significant variations in fat content among species (Table 1) and even within the same species, depending on dietary substrate and environmental conditions [272, 273]. Among the analyzed species, *Tenebrio molitor* exhibits the highest lipid levels, ranging from 6.1% to 58.2%, followed by *Hermetia illucens* (4.9%–49.0%), *Zophobas morio* (17.6%–46.3%), and *Bombyx mori* (13.0%–35.7%) [54, 64, 81, 104]. In contrast, *Musca domestica* and *Acheta domesticus* contain lower fat levels, ranging from 1.5% to 30.1%, depending on diet and developmental stage [72, 74, 92]. Factors such as insect taxonomic order, species, rearing environment, and dietary substrate significantly influence the fatty acid profile, as indicated in Table 4 [44, 203, 274, 275]. In general, *H. illucens* stands out for its high content of SFAs, with lauric acid (C12 : 0) predominating, representing up to 62.75 g/100 g of total lipids. It also has moderate concentrations of palmitic acid (C16 : 0), ranging from 1.03 to 21.90 g/100 g, and stearic acid (C18 : 0), between 0.92 and 6.90 g/100 g [63, 117]. This profile differs from that of other species such as *M. domestica* and *T. molitor*, which have higher concentrations of MUFAs and PUFAs [152, 202].

Among MUFAs, oleic acid (C18 : 1n–9) is present in significant proportions. *Tenebrio molitor* can contain up to 48.7 g/100 g, while *Zophobas morio* ranges between 27.75 and 122.49 g/100 g, and *Bombyx mori* can reach 29 g/100 g [132, 141, 200]. This profile is advantageous for aquaculture feeds, as oleic acid contributes to maintaining cellular integrity and fish energy metabolism [276]. PUFAs are also found in considerable amounts in edible insects, particularly linoleic acid (C18 : 2n–6) and α-linolenic acid (C18 : 3n–3) [93]. Linoleic acid is present at high levels in *A. domesticus*, reaching up to 41.39 g/100 g, and in *Z. morio*, with values up to 71.05 g/100 g. On the other hand, α-linolenic acid is found at lower concentrations, ranging from 0.03 to 13.10 g/100 g [44, 123, 129, 209]. Despite the significant presence of PUFA in edible insects, one challenge in their application to aquaculture diets is the low concentration of long-chain omega-3 fatty acids such as EPA and DHA, which are essential for marine fish health. However, strategies such as modifying insect diets can enhance these levels, making them a nutritionally more complete alternative for aquaculture.

### 1.11. Vitamins and Minerals

Edible insects have a range of vitamins. B-complex vitamins are widely found in the analyzed insects, with riboflavin (B2) standing out, present in high concentrations in *H. illucens* (5.00–24.74 mg/100 g) and *A. domesticus* (0.09–11.07 mg/100 g), as well as niacin (B3), which can reach 19.36 mg/100 g in *A. domesticus* and 15.20 mg/100 g in *B. mori* [62, 92, 104]. The presence of vitamin B12, essential for metabolic processes, is most notable in *T. molitor* (0.47–0.56 mg/100 g) and *Z. morio* (0.42–0.99 mg/100 g), whereas *H. illucens* and *M. domestica* do not present detectable amounts of this nutrient [73, 81]. Additionally, *A. domesticus* exhibits the highest reported levels of vitamin E (1.15–332.06 mg/100 g), a crucial antioxidant for cellular health and membrane lipid stability [92]. Although insects have a rich nutritional profile in micronutrients, the bioavailability of these compounds may be affected by factors such as the presence of chitin and the processing method of the insect meal. Techniques such as enzymatic hydrolysis and fermentation have been studied to improve nutrient absorption and maximize their benefits in fish diets [261]. Therefore, including insects in aquafeed formulations can significantly contribute to the supply of essential vitamins and minerals, provided that their composition is strategically balanced to meet the nutritional requirements of different fish species.

Minerals play an essential role in fish nutrition, acting in processes such as energy metabolism, growth, bone development, osmotic balance, and immune response. They are divided into macrominerals, which are required in relatively high amounts in the diet (g/kg), and microminerals, which are needed in lower concentrations (mg/kg or μg/kg) [277]. Edible insects are a significant source of minerals such as Ca, P, K, Na, Mg, Fe, Zn, Mn, and Cu [155, 278–280]. However, the bioavailability of these minerals can vary considerably among insect species and be influenced by diet and developmental stage [277, 281].

Fish absorb Ca and P from the surrounding aquatic environment through the gills, gastrointestinal tract, and skin. However, the gills are the primary site of Ca absorption [282], while food is the main source of P for both marine and freshwater fish, as these environments are naturally low in phosphate [283]. The Ca requirement in fish is influenced by dietary factors (e.g., bioavailability and P level in the diet), absorption from water, and species variations [254, 284]. The dietary Ca requirement is relatively low for most species (< 340 mg/100 g), as much of their need is met by absorption through the gills in freshwater fish and by seawater ingestion in marine species [254, 284–286]. However, tilapia reared in water with a low Ca concentration (< 1 mg Ca L^−1^) required 0.45% and 0.7% Ca in the diet [287], as indicated in Table 3.

The P requirement in fish is more significant since its primary acquisition route is through diet. The dietary P requirements vary widely among different species, such as Atlantic salmon (*Salmo salar*) (600–1000 mg/100 g) [288–290]; rainbow trout (*Oncorhynchus mykiss*) (340–800 mg/100 g) [285, 291, 292]; Nile tilapia (*Oreochromis niloticus*) (650–860 mg/100 g) [293, 294]; and common carp (*Cyprinus carpio*) (600–700 mg/100 g) [285]. For most aquaculture species, the ideal Ca-to-P ratio ranges from 1 : 1 to 1 : 2, which is crucial for ensuring proper growth and preventing nutritional deficiencies [295]. Among the studied insects, *H. illucens* has the highest Ca concentration (120–3570 mg/100 g) [62, 117], making it a valuable source of this essential mineral for fish growth and development [296]. However, its high P content (100–4800 mg/100 g) [146, 147] requires careful balance to avoid interference in nutrient absorption and energy metabolism [297].

K, Na, and chloride (Cl) are the primary electrolytes in fish plasma and are crucial for osmoregulation, acid–base balance, and nerve function [298]. Most fish species can obtain these electrolytes from their aquatic environment, reducing their dietary requirement [254]. However, in intensive farming systems where water quality fluctuates, dietary supplementation may be necessary. K is the most abundant intracellular cation and plays an essential role in enzyme activation, osmoregulation, and nerve impulse transmission [282]. The dietary K requirement for fish ranges from 0.5% to 1.5%, depending on the species and rearing conditions [299]. Na is the primary extracellular cation, regulating blood pressure and water balance, with dietary requirements varying between 0.15% and 0.5% [300]. Cl is essential for the production of hydrochloric acid (HCl) in the stomach and for maintaining acid–base equilibrium, with an estimated requirement of 0.15%–0.4% in fish diets [301].

Dietary Mg is considered the primary source for fish growth and development [254]. Mg plays a role in nerve transmission, enzymatic activation, and is an important intracellular energy molecule (ATP) [295]. The dietary Mg requirement in fish varies between 150 and 500 mg/kg of feed [254]. Mg deficiency can result in reduced growth, muscular tremors, and increased mortality [301]. Insects contain varying amounts of Mg, depending on their species and diet, with *H. illucens* larvae having Mg levels ranging from 50 to 300 mg/100 g [117, 129]. This indicates their potential as a source of this essential mineral in aquafeeds.

Fe is one of the most studied trace minerals, being present in all vertebrate body cells [254, 295]. It is fundamental for oxygen transport (hemoglobin and myoglobin) and cellular metabolism [302]. The dietary Fe requirement varies by species and ranges from 30 to 150 mg/kg of diet [299, 303]. Deficiency leads to anemia, reduced growth, and impaired immune function [304]. Insects are considered a good source of Fe, with *H. illucens* larvae containing 3–10 mg/100 g [155, 305], making them a viable alternative to traditional sources such as FM and mineral supplements.

Zn is an essential cofactor for numerous enzymes involved in protein and DNA synthesis, growth, and immune response [254]. The dietary Zn requirement for fish ranges from 15 to 150 mg/kg [306, 307]. Deficiencies can lead to growth retardation, cataracts, fin and skin erosion, and reduced immune function [254, 299]. Insects such as *H. illucens* and *T. molitor* contain significant Zn levels (5–30 mg/100 g) [305, 308], reinforcing their potential as a dietary Zn source in fish diets.

Mn and Cu are required in trace amounts for enzyme function, antioxidant defense, and bone formation [254]. The dietary requirement for Mn in fish varies between 2 and 20 mg/kg [254, 303] while Cu requirements range from 3 to 10 mg/kg [299]. Deficiencies can cause growth retardation, skeletal deformities, and reduced enzyme activity [254]. Insects contain variable concentrations of these minerals, with *H. illucens* larvae reported to have Mn levels of 0.5–5 mg/100 g and Cu levels of 0.5–10 mg/100 g [231, 305].

Overall, edible insects, particularly *H. illucens* and *T. molitor*, represent a promising alternative source of essential minerals for aquafeeds. Their mineral composition is influenced by diet and rearing conditions, requiring careful formulation to optimize bioavailability and meet fish nutritional requirements [277, 309]. Future studies should focus on mineral digestibility and interactions with other nutrients to maximize the benefits of insect-based feeds in aquaculture production.

### 1.12. Edible Insects as Feed for Fish

Insect farming for the feed industry has grown significantly worldwide [252]. Due to their remarkable nutritional profile, as previously discussed, the use of insects in fish feed production is considered one of the most sustainable and economically viable alternatives [46, 310]. Numerous studies have demonstrated that insect meal can be successfully incorporated into the diets of various freshwater and marine fish species without compromising growth or health [184, 229, 311]. Furthermore, processing technologies such as drying, fat extraction, and enzymatic hydrolysis have been applied to enhance the digestibility and nutritional quality of insect meal [312].  Table 6 summarizes a selection of studies examining the effects of six key insect species used as FM replacers in different aquaculture species.

### 1.13. Black Soldier Fly (*Hermetia illucens*)

The inclusion of *Hermetia illucens* meal in the diet of various fish species has been widely studied, with positive results at different levels of FM replacement. When used at proportions between 20% and 64% for omnivorous and/or freshwater fish, no negative effects were observed on growth, feed conversion, or survival rate, and hematological indices remained stable [313, 315–317, 319]. In the case of carnivorous species, replacement levels of up to 40% preserved nutrient digestibility in rainbow trout (*Oncorhynchus mykiss*) [123], although they reduced PUFA deposition in the fillet [116]. For European seabass (*Dicentrarchus labrax*), replacement levels of up to 19.5% did not negatively affect growth or digestibility [320]. The same was observed with the total replacement of FM in the diet of Atlantic salmon (*Salmo salar*) [321]. Although *H. illucens* meal has great potential as an alternative ingredient for aquafeeds, excessively high inclusion levels may compromise growth or intestinal integrity in some species. Therefore, the optimal replacement levels should be adjusted according to the physiological and nutritional requirements of each species to ensure a balance between sustainability and productive performance.

### 1.14. House Fly (*Musca domestica*)

The use of *M. domestica* larva meal has been reported in various species. In freshwater fish such as the African catfish (*Clarias gariepinus*), the replacement of up to 75% of FM with *M. domestica* larvae resulted in higher weight gain and reduced feed costs, with no negative impacts on zootechnical performance [322, 325]. Similar results were observed in hybrid catfish (*Clarias gariepinus* × *Heterobranchus longifilis*), where diets containing 21% *M. domestica* promoted the best growth rates and immunological parameters, highlighting its potential as a protein supplement [326]. Supplementation with low levels of pupae meal (0.5% and 5%) resulted in superior growth and improved immunity in marine species such as *Pagrus major*, as evidenced by increased phagocytic activity of peritoneal leukocytes [323]. For Nile tilapia (*Oreochromis niloticus*), moderate inclusion levels of *M. domestica* maintained growth comparable to the control. However, total replacement of FM resulted in lower feed efficiency, indicating that the suitability of this ingredient may vary depending on the species and inclusion level [324]. Thus, the potential of *M. domestica* as an alternative protein source is evident, especially considering that its nutritional quality can be optimized for specific applications in aquaculture [331] to ensure balanced diet formulation for fish.

### 1.15. Superworm (*Zophobas morio*)

Dietary inclusion of up to 30% *Zophobas morio* meal maintained protein digestibility, feed conversion, and growth in marine fish such as cobia (*Rachycentron canadum*) [333]. Similarly, in gilthead seabream (*Sparus aurata*), replacing 10%–30% of FM improved feed conversion and strengthened the immune response without compromising growth [336]. Additionally, a 20% inclusion level had immunomodulatory effects, while 30% increased nitric oxide production, potentially enhancing disease resistance [332]. *Z. morio* meal has also shown promise in rainbow trout (*Oncorhynchus mykiss*) diets, where 25% inclusion resulted in better growth and feed conversion, with no adverse effects on the histology of the liver, kidney, or intestine, indicating good safety for the species [334]. In goldfish (*Carassius auratus*), higher *Z. morio* inclusion levels in the diet improved digestion and enhanced carotenoid deposition in the skin and muscles, suggesting its potential as a natural pigmentation source [358]. Despite these positive results, some limitations have been observed regarding protein and lipid digestibility in red tilapia (*Oreochromis* sp.), reducing feed efficiency [131], highlighting the importance of using this ingredient at moderate levels.

### 1.16. Mealworm (*Tenebrio molitor*)

Recent research has provided consistent evidence highlighting *Tenebrio molitor* as an innovative protein ingredient for the partial replacement of FM in aquafeeds [135, 184]. Several studies have evaluated its inclusion in the diets of rainbow trout (*Oncorhynchus mykiss*) [248, 343, 346], European seabass (*Dicentrarchus labrax*) [337, 340], and Nile tilapia (*Oreochromis niloticus*) [317, 324], indicating that the optimal replacement level depends on the feeding habits and farming conditions of each species [230], as presented in Table 6. In carnivorous species such as rainbow trout (*O. mykiss*), European seabass (*D. labrax*), and sea trout (*Salmo trutta m. trutta*), replacement levels of up to 40% did not affect feed intake, growth [337, 340, 343], or gastrointestinal enzyme activity [344], but they did reduce plasma cholesterol and beneficial fatty acid levels, such as EPA and DHA.

In freshwater fish, the effects of replacing FM with *T. molitor* vary according to the species' tolerance. In grass carp (*Ctenopharyngodon idellus*), levels of up to 25% improved growth and protein efficiency, but higher replacements led to oxidative stress and impaired liver function, indicating a safe inclusion limit [338]. In Nile tilapia (*O. niloticus*), up to 50% inclusion maintained protein digestibility and muscle composition but reduced growth, possibly due to the presence of chitin and alterations in lipid metabolism [261]. Conversely, in African catfish (*Clarias gariepinus*), replacement levels of up to 40% had no adverse effects on performance, and even at 80%, growth remained acceptable, suggesting that this species has a higher tolerance to insect-based diets [341]. Overall, replacing FM with *T. molitor* can be a viable and beneficial strategy for various aquaculture species, provided that inclusion levels are adjusted to prevent negative impacts on growth and metabolism.

### 1.17. House Cricket (*Acheta domesticus*)

Recent studies have shown that house cricket (*Acheta domesticus*) can replace FM in diets for Nile tilapia (*Oreochromis niloticus*), although the inclusion rate affects fish zootechnical performance. Perera et al. [347] reported that the complete replacement of FM with house cricket resulted in lower weight gain and a poorer feed conversion ratio, while fish survival was not compromised. Conversely, Fawole et al. [326] indicated that including 35% house cricket in the diet led to growth comparable to the commercial control, suggesting that this ingredient could be a viable alternative for tilapia feed formulation. Lee et al. [349] evaluated the partial and total replacement of commercial diet protein with *Acheta domesticus* meal combined with rice bran in the nutrition of red tilapia (*Oreochromis* sp.). The results showed that a diet containing 60% *A. domesticus* meal + 40% rice bran promoted the best growth and survival rates, comparable to the commercial diet. However, diets with higher replacement levels (>70%) resulted in reduced growth and hepatic alterations, highlighting a potential digestive limitation or the presence of antinutritional compounds.

### 1.18. Silkworm (*Bombyx mori*)

The use of *Bombyx mori* in studies with omnivorous and freshwater species, such as Nile tilapia (*Oreochromis niloticus*) and walking catfish (*Clarias batrachus*), indicate that FM replacement levels of up to 66.6% do not negatively affect growth, feed conversion, or protein efficiency [351, 355]. In Gift tilapia (*O. niloticus*), 40% inclusion led to significant improvements in weight gain, feed efficiency, and hematological parameters [354]. For carnivorous species like rainbow trout (*Oncorhynchus mykiss*) and African catfish (*Clarias gariepinus*), partial replacement of FM with silkworm pupae meal yielded mixed results. While up to 10% inclusion in rainbow trout maintained growth and feed conversion efficiency [357], higher levels negatively impacted performance. In African catfish, optimal growth and feed efficiency were achieved at 50% inclusion, but full replacement led to reduced performance [353]. A different approach was tested in mirror carp (*Cyprinus carpio var. specularis*), where fermented silkworm pupae meal was included at levels up to 160 g/kg. While up to 40 g/kg replacement did not impair growth or health, higher inclusion levels resulted in reduced weight gain, feed utilization, and altered lipid metabolism [356].

### 1.19. Challenges and Future Perspectives

#### 1.19.1. Integrated Development of Insect Meals for Sustainable Aquaculture

Although insect farming has long been practiced in animal nutrition, its large-scale industrial application in aquafeeds remains incipient, limiting its capacity to meet the growing protein demands of global aquaculture or to significantly reduce feed costs [359]. Scaling up production will require substantial investments in infrastructure, automation, and processing technologies to improve cost-efficiency and ensure a consistent and high-quality supply [360]. While FM and plant-based ingredients still dominate aquafeeds, their long-term viability is increasingly constrained by land and water limitations, rising input costs, and nutritional shortcomings such as amino acid imbalances and reduced palatability [87, 361, 362]. In this context, insect meals emerge as a promising alternative that could offset some of the limitations of both FM and plant protein sources.

Promising advances are already being observed in salmonid aquaculture, where insect meals have received regulatory approval in the European Union (EU) and are being incorporated into commercial feeds. Industry reports suggest that replacing traditional protein sources with insect-based ingredients does not compromise growth performance or feed conversion in high-value species like salmon [168, 260, 363]. Moreover, consumer acceptance in countries such as Norway has been favorable, reinforcing the potential for market penetration in premium aquaculture sectors [364].

However, broader commercialization across diverse aquaculture systems still faces major hurdles. Current production volumes of insect meals remain insufficient to meet the demands of species such as tilapia or shrimp, which dominate global aquaculture by volume. For example, projections for the European market in 2022 estimated that five producers would each generate around 20,000 tonnes of insect meal per year, only about two-thirds of the soybean concentrate used by a single major feed manufacturer like Skretting Norway [168, 260, 363, 365–370]. This highlights the need for strategic partnerships between insect producers and large-scale feed companies to overcome supply limitations and facilitate integration into mainstream aquafeed formulations.

One of the most compelling aspects of insect farming is its compatibility with circular economy principles. Agri-tech companies in Southeast Asia have begun to implement integrated production systems that transform agro-industrial waste into high-value insect biomass and organic fertilizers, thereby enhancing the ecological rationale of insect-based feeds [364]. These systems contribute not only to protein security but also to waste reduction and nutrient recycling, aligning insect farming with the broader goals of the blue economy.

Despite these advances, several technical and environmental challenges remain. Scaling up insect meal production demands improvements in energy efficiency, logistics, and life-cycle sustainability metrics to validate its long-term ecological benefits [52]. Moreover, the immunonutritional properties of insect meals, which support fish health and growth, are promising but require further optimization through refined amino acid balancing and the mitigation of antinutritional factors. Standardization of rearing substrates and assurance of microbiological safety are also critical steps toward consistent product quality and regulatory compliance. Taken together, these developments suggest that insect meals are no longer a distant prospect but a near-term reality in select aquaculture markets. Nevertheless, the transition from niche adoption to widespread implementation will depend on targeted innovation, coordinated supply chain scaling, and the establishment of robust standards for quality and sustainability. Future research and policy should focus not only on performance metrics but also on systemic integration, enabling insect-based feeds to contribute meaningfully to a more resilient and sustainable aquaculture sector.

#### 1.19.2. Standardization of Rearing Substrates and Microbiological Safety of Insect Meals

One of the most significant challenges to the industrial scaling of insect meals for aquaculture is the lack of standardization in rearing substrates. While the use of diverse organic materials aligns with circular economy principles and enhances the environmental appeal of insect farming, it also introduces substantial variability in the nutritional quality of the final product. Substrate composition directly affects critical parameters such as crude protein levels, amino acid profiles, and digestibility coefficients, factors that are essential for the formulation of nutritionally consistent and efficient aquafeeds [359]. This inconsistency limits product standardization, undermines reproducibility, and poses a barrier to broader adoption by the feed industry.

Microbiological safety presents a parallel concern. Insects can harbor or transmit pathogenic microorganisms via their gut microbiota or external surfaces, especially when reared under suboptimal sanitary conditions [371–373]. Although Commission Regulation (EU) 2017/893 considers unprocessed insect biomass to present a hazard level similar to or lower than other animal-derived proteins, the potential for contamination by pathogens such as *Salmonella*, *Campylobacter*, and *Escherichia coli* necessitates stringent hygiene controls throughout the production chain [374, 375]. Preventive measures, including the application of HACCP protocols, sanitary substrate selection and treatment, and rigorous environmental management, are crucial for maintaining microbiological integrity.

In addition to biological hazards, the risk of chemical contamination must also be addressed. When insects are reared on agro-industrial or municipal waste streams, they may be exposed to pollutants such as heavy metals, pesticides, or dioxins. Although the short life cycle of species commonly used in feed production tends to reduce the extent of bioaccumulation [309], systematic monitoring and strict regulatory oversight remain indispensable. Ensuring the chemical safety of insect-derived ingredients is particularly important in aquaculture, where feed quality directly influences animal health and food safety outcomes.

Furthermore, while most insect-specific pathogens are not zoonotic and pose minimal direct risk to vertebrates, their impact on colony health and production efficiency cannot be overlooked. Maintaining pathogen-free colonies is essential not only for productivity but also for ensuring consistent yields and product quality. In summary, the dual need for nutritional consistency and microbiological safety highlights the importance of developing standardized rearing protocols and implementing robust quality assurance systems. Addressing these issues will be pivotal for the long-term credibility and regulatory acceptance of insect meals in aquaculture, especially as the sector moves toward greater integration into global feed supply chains.

#### 1.19.3. Regulatory Frameworks and Feed Safety

The regulatory framework for insect-based feeds has undergone substantial evolution, particularly in the EU. Initially, progress was stymied by Regulation (EC) Number 999/2001, introduced in response to the Bovine Spongiform Encephalopathy (BSE) crisis, which banned processed animal proteins (PAPs) in livestock feed and effectively excluded insect meals from the market [376, 377]. This changed with Regulation (EU) 2017/893, which authorized the use of nonruminant PAPs and approved seven insect species for inclusion in aquafeeds: *Hermetia illucens*, *Musca domestica*, *Tenebrio molitor*, *Alphitobius diaperinus*, *Acheta domesticus*, *Gryllodes sigillatus*, and *Gryllus assimilis* [378, 379]. That same year, Regulation (EU) 2017/1017 further allowed terrestrial invertebrates, live or processed, to be used as feed materials [376].

Subsequent advancements included Regulation (EU) 2021/1372, extending insect PAP use to poultry and swine feeds [380], and Regulation (EU) 2021/1925, which added *Bombyx mori* to the list of authorized species [381, 382]. Despite these milestones, several barriers remain. There are still no insect-specific good manufacturing practices (GMPs), and producers must rely on general food and feed legislation [375]. Moreover, insects can only be reared on substrates deemed “feed grade,” such as plant-based materials and selected animal byproducts (e.g., FM, dairy residues, and nonruminant blood products) [383]. While these constraints aim to ensure safety, they also restrict innovation, particularly regarding the use of organic waste or regionally abundant by-products.

The European Food Safety Authority (EFSA) has played a central role in shaping policy. Its 2015 scientific opinion concluded that when reared on approved substrates, insects pose microbiological risks similar to those of other animal-derived proteins [384, 385]. This assessment laid the groundwork for Regulation (EU) 2015/2283 on novel foods, which came into force in 2018. Under this framework, insect-based products intended for human consumption must receive prior approval following EFSA safety assessments and meet labeling requirements, including allergen disclosures [379, 380, 385].

In recent years, the number of approved insect-based foods has grown. Regulation (EU) 2021/882 authorized dried *T. molitor* as the first insect novel food [377], followed by *Locusta migratoria*, *Acheta domesticus*, and more recently, freeze-dried or frozen forms of *A. diaperinus*. Applications for *H. illucens*, *G. sigillatus*, and *Apis mellifera* remain under EFSA review [379]. However, complex approval procedures and persistent consumer safety concerns have hindered market expansion [385].

Outside the EU, regulatory approaches remain fragmented. In the United States, insect feed is regulated jointly by the FDA and AAFCO. *H. illucens* is approved for aquafeeds, and crickets were authorized for pet food in 2018. Canada requires registration of insect-based ingredients, with *H. illucens* approved for aquaculture and poultry feeds. Australia and New Zealand allow species like *Acheta domesticus* and *Zophobas morio*, provided they comply with general safety standards [386]. In contrast, regulation in Asia is inconsistent: China permits insects as feed additives under generic rules, South Korea lacks dedicated legislation, and North Korea prohibits insect-based feed entirely [387, 388].

This regulatory heterogeneity complicates global trade and obstructs the harmonization of international standards for insect-based feed products. Consumer acceptance adds another layer of complexity. Although permitted in aquafeeds across the EU, acceptance varies widely between countries, with relatively higher receptivity reported in Belgium and Italy [389–392]. For effective and widespread implementation of insect-based feed regulations, two elements are essential: robust safety assessments and transparent, science-driven communication strategies that engage producers, retailers, and consumers alike [376, 393]. These will be key to bridging regulatory gaps and fostering public trust in insect-derived feed ingredients. However, regulatory progress alone is not sufficient to ensure market success—consumer perception plays an equally decisive role in the integration of insect-based feeds.

#### 1.19.4. Consumer Acceptance and Perception

Although regulation has advanced and interest from the aquafeed industry continues to grow, consumer acceptance remains one of the most significant barriers to the widespread adoption of insect-based ingredients. In many Western societies, the practice of entomophagy is not part of culinary traditions, leading to low acceptance due to general unfamiliarity with insect consumption [380, 385]. While insect consumption is culturally embedded in several Asian, African, and Latin American countries, western consumers often exhibit aversion, not only to direct consumption but also to products derived from insect-fed animals, including fish [388].

From a psychological standpoint, food neophobia and disgust are among the most influential factors shaping consumer responses [394, 395]. These reactions are closely associated with rejection of insect-based products, whereas individuals with lower levels of these traits tend to be more open to experimentation [396]. In contrast, environmental awareness and sustainability claims can positively influence attitudes, particularly when insect-fed animals are framed as contributing to reduced land use, water consumption, and greenhouse gas emissions [397]. Nonetheless, this positive perception does not always translate into purchasing behavior, revealing a gap between interest and actual willingness to buy [392].

Empirical studies from European countries, including Italy, Greece, Spain, and France, indicate that informed consumers are more accepting of insect-fed fish. For instance, consumers in Greece and Italy expressed higher willingness to buy gilthead seabream fed with insect meal, especially when sustainability benefits were emphasized [397, 398]. Men and younger individuals consistently showed greater openness. In Greece, a survey at an aquaculture conference found that 71% of participants would consume insect-fed fish, primarily due to environmental motivations. Similarly, in Spain, consumers associated these products with sustainability and were even willing to pay a premium, although taste remained a concern [399]. Studies further show that consumers prefer indirect entomophagy—such as eating fish raised on insect-based diets—over direct consumption of insect products [400]. In France, participants informed about the environmental downsides of FM and the benefits of insect meal exhibited greater acceptance of insect-fed trout [401]. These findings underscore the importance of targeted communication and transparency in shaping perception.

Perceived nutritional value and food safety are also crucial. Products labeled as rich in high-quality protein and micronutrients are generally better received, especially when accompanied by transparent labeling and third-party certifications [389, 394]. Conversely, concerns about allergens and health risks continue to hinder acceptance. Sensory attributes such as appearance and taste also affect purchasing decisions. While taste is a significant factor—especially for consumers unfamiliar with insect-based products—studies show that replacing conventional protein with insect meal often has limited impact on flavor [402]. Clear communication of these findings may help mitigate disgust and improve perception [403]. Socio-demographic factors such as age, gender, education, and diet further influence attitudes. Flexitarians and vegetarians tend to be more receptive to novel protein sources, whereas habitual meat eaters are often more resistant [380, 404]. Higher education levels correlate with greater openness, likely due to increased exposure to scientific information. In Norway, where insect meal is already implemented in salmon farming, consumers appear more favorable toward this practice, especially when it is framed within narratives of sustainability and supported by robust regulatory frameworks [390]. These insights highlight the value of context-specific communication strategies, aligned with local values and expectations. Initiatives embedded in the blue economy could reinforce this narrative by linking insect-based feeds to ecological and socioeconomic benefits.

Lastly, economic feasibility remains a key constraint. Insect-based meals are still more expensive than FM, with prices ranging from €2,000 to €5,000 per ton depending on species and production method [252, 405]. While this limits commercial viability, rising FM costs may eventually shift the balance in favor of insect proteins [396]. In summary, although scientific and environmental arguments provide a strong rationale for adopting insect-based aquafeeds, long-term consumer engagement will depend on culturally tailored messaging, transparent labeling, and education. Promoting insect-fed fish as safe, nutritious, and environmentally responsible is essential for gaining broader market acceptance and advancing circular economy principles. Ultimately, the future of insect-based aquafeeds depends not only on regulatory and technological progress but also on the industry's capacity to build trust and communicate value effectively.

## 2. Conclusion

Research on the use of edible insects in aquafeeds continues to expand, reinforcing their potential as a viable and effective alternative to conventional ingredients. Their growing recognition as a key component of modern aquaculture highlights their ability to enhance efficiency and resilience in fish production systems. The continued development of this sector represents a crucial step toward reducing dependance on marine-derived ingredients while preserving the nutritional integrity of aquafeeds. As technological advancements and regulatory frameworks evolve, edible insects could play a pivotal role in shaping the future of aquaculture, offering a sustainable and nutritionally rich alternative to fish nutrition. Moreover, it is important to note that edible insects are fully integrated into the circular economy. They can be partially reared on agro-industrial byproducts, whose destination is often landfilling, contributing to environmental degradation. This bioconversion process results in valuable products such as protein-rich flours, lipid-rich oils, and bioactive compounds. Furthermore, insect frass serves as an effective organic fertilizer, while their exoskeletons provide a source of chitin—a nitrogen-containing polysaccharide with diverse applications across multiple industries. In conclusion, the incorporation of edible insects in aquaculture aligns with global efforts to protect the environment and create a more sustainable future.

## Figures and Tables

**Table 1:** Nutritional value (g/100 g dry weight) of most common insects used as fish feed.

Insect species	Protein	Fat	Fiber	Ashes	References
HI	29.9–56.1	4.9–49.0	5.2–11.4	4.1–28.4	[62–71]
MD	8.8–76.2	1.5–30.1	8.7–10.1	2.6–12.8	[72–79]
ZM	18.1–48.1	17.6–46.3	4.8–11.3	0.6–4.89	[54, 71, 80–86]
TM	18.8–76.2	6.1–58.2	5.2–10.3	0.8–8.1	[54, 71, 81, 82, 85–91]
AD	13.1–76.1	1.5–43.9	1.0–28.0	0.6–9.1	[88, 92–100]
BM	21.5–80.0	13.0–35.7	3.5–14.0	1.0–1.4	[101–110]

Abbreviations: AD, *Acheta domesticus*; BM, *Bombyx mori;* HI, *Hermetia illucens*; MD, *Musca domestica*; TM, *Tenebrio molitor*; ZM, *Zophobas morio*.

**Table 2:** Amino acid composition (g/100 g protein) of different insect species.

Amino acid	Insect species					
	HI	MD	ZM	TM	AD	BM
**Essential amino acids (gAA/100 g protein)**						

Valine	2.29–6.79	1.30–3.61	1.03–7.37	1.10–6.14	1.07–5.91	0.38–5.60
Isoleucine	1.76–6.79	1.46–3.50	0.93–6.97	0.83–7.38	0.93–4.45	0.30–5.70
Leucine	2.67–7.83	1.71–6.35	1.91–7.68	1.40–10.60	1.11–9.75	0.49–8.30
Lysine	2.30–7.40	2.23–6.90	1.03–7.43	1.02–6.99	1.10–5.40	0.40–7.50
Threonine	1.42–4.50	1.35–7.60	0.78–5.15	0.66–4.57	0.74–3.65	0.29–6.15
Phenylalanine	1.35–7.76	1.56–10.20	0.68–5.41	0.65–5.48	0.65–3.38	0.27–5.10
Methionine	0.60–2.12	0.62–2.60	0.21–2.02	0.24–3.16	0.30–1.40	0.13–4.60
Histidine	0.98–4.80	0.52–5.10	0.60–5.07	0.56–4.67	0.48–2.25	0.24–6.63
Tryptophan	0.54–0.63	0.85–2.39	0.18–0.50	0.15–1.00	0.13–1.43	0.07–1.50

**Nonessential amino acids (gAA/100 g protein)**						

Tyrosine	1.71–6.71	1.04–4.90	1.37–12.12	1.37–10.08	1.00–3.48	0.29–5.61
Arginine	1.80–6.20	0.70–5.80	0.96–8.53	0.97–7.10	1.25–6.10	0.39–6.80
Aspartic acid	3.30–10.30	3.47–8.50	1.58–9.90	1.52–9.74	1.72–7.75	0.61–10.90
Glutamic acid	4.58–13.30	5.57–15.30	2.42–14.40	2.11–13.09	2.15–10.45	0.96–14.90
Serine	1.55–4.88	1.27–3.30	0.92–4.88	0.96–4.28	1.02–2.87	0.34–5.04
Glycine	0.12–6.15	0.90–5.35	0.95–5.72	0.16–6.58	1.04–3.60	0.56–7.11
Alanine	2.50–8.21	2.71–4.40	1.43–7.43	1.64–6.29	1.37–8.85	0.42–6.10
Cysteine	0.02–0.76	1.48–2.17	0.25–0;36	0.16–1.51	0.40–0.80	0.54–1.40
Proline	2.30–6.68	1.10–3.17	1.08–10.60	1.21–11.27	1.15–3.54	0.32–7.00
References	[62, 63, 116, 117–122, 123]	[75–77, 124–128]	[80, 81, 83, 123, 129–131]	[81, 88, 129, 3, 91, 132–134]	[88, 98, 123, 129, 135–138]	[129, 139–145]

Abbreviations: AD, *Acheta domesticus*; BM, *Bombyx mori;* HI, *Hermetia illucens*; MD, *Musca domestica*; TM, *Tenebrio molitor*; ZM, *Zophobas morio*.

**Table 3:** Vitamin and mineral requirements (mg/100 g protein) of different fish species.

Micronutrient	Insect species					
	HI	MD	ZM	TM	AD	BM
**Vitamins (mg/100 g protein)**						

Vitamin A (beta carotene)	NR	NR	<0.02	<0.02	0.02–3.34	NR
Vitamin B1 (thiamine)	1.98–2.00	NR	0.06–0.17	0.06–0.36	0.02–1.66	0.07–1.91
Vitamin B2 (riboflavin)	5.00–24.74	NR	0.75–1.12	0.37–1.61	0.09–11.07	2.23–5.43
Vitamin B3 (niacin)	0.19–0.37	NR	3.23–3.53	0.07–5.64	0.11–19.36	2.20–15.20
Vitamin D	NR	NR	4.00–25.60	4.00–25.60	4.00–17.15	<0.010
Vitamin E	3.30–24.80	NR	0.77–16.30	0.50–0.99	1.15–332.06	5.14–9.89

**Minerals (mg/100 g protein)**						

Calcium (Ca)	120–3570	410–1460	31.9–78.8	16.90–2930	27.5–1868.5	30.73–158
Potassium (K)	170–1700	220–1270	493.19–1065.1	741.7–1008.3	240.8–1598.6	<1826.59
Phosphorus (P)	100–4800	120–2400	547.11–628.47	277.00–1270	12.69–1806	474–1369.9
Sodium (Na)	50–1560	507–824.37	104.10–187.7	48.90–181.97	53.1–863.3	140.2 −328.08
Magnesium (Mg)	100–560	820–1150	39.20–124.55	60.60–308.63	19.3–622	207–287.96
Zinc (Zn)	14.21–10300	1.06–32.53	2.50–13.92	4.40–16.05	2.17–24.4	17.75–23
Iron (Fe)	56.09–19100	4.65–27.52	3.20–11.58	2.06–6.66	0.97–16.86	2.60–26
Manganese (Mn)	10.10–16600	3.70–34.85	0.50–1.20	0.36–2.19	0.46–9.53	0.71–1.04
Copper (Cu)	10–1500	0.25–1.81	0.50–1.42	0.61–2.75	0.51–5.08	0.15–1.04
References	[62, 146, 147, 117, 119, 148, 149, 70, 73, 150]	[77, 79, 126, 151, 152]	[81, 129, 153, 154, 71, 84]	[71, 81, 129, 133, 154–160]	[94–96, 98, 92, 161, 162, 129, 157, 163, 137]	[101, 104, 141, 142, 144, 164–166]

Abbreviations: AD, *Acheta domesticus*; BM, *Bombyx mori*; HI, *Hermetia illucens*; MD, *Musca domestica*; MUFAs, monounsaturated fatty acids; NR, not reported; PUFAs, polyunsaturated fatty acids; SFAs, saturated fatty acids; TM, *Tenebrio molitor*; ZM, *Zophobas morio*.

**Table 4:** Fatty acid composition (g/100 g of total fatty acids) of different insect species.

Fatty acid (g/100 g)	Insect species					
	HI	MD	ZM	TM	AD	BM
**SFA**						

Caprylic (C8 : 0)	NR	NR	0.22–1.80	0.01–0.10	NR	0.062–0.15
Capric (C10 : 0)	0.81–14.30	NR	0.10–0.40	0.05–0.10	<0.10	<0.16
Lauric (C12 : 0)	7.50–62.75	0.18–1.54	0.03–0.46	0.10–2.90	0.02–0.15	0.20–0.38
Myristic (C14 : 0)	2.30–10.70	2.56–9.23	0.09–2.94	0.20–6.57	0.009–1.13	0.10–10.0
Pentadecanoic (C15 : 0)	0.13–14.38	0.86–1.06	0.20–0.64	0.06–7.10	0.01–1.10	<0.4
Palmitic (C16 : 0)	1.03–21.90	20.28–38.01	17.29–98.11	0.02–19.76	1.54–27.66	0.41–28.60
Margaric (C17 : 0)	0.13–0.26	<0.90	0.58–2.00	0.03–20.00	0.02–0.58	0.05–0.20
Stearic (C18 : 0)	0.92–6.90	2.32–11.79	2.20–20.08	0.03–6.60	0.58–17.35	0.40–63.0
Arachidic (C20 : 0)	0.02–0.03	0.09–0.14	0.17–21.90	0.08–25.10	0.04–1.25	0.10–0.13

**MUFA**						

Palmitoleic (C16 : 1)	0.80–14.10	5.59–28.59	0.44–4.04	0.09–5.64	0.09–1.35	0.10–31.0
Heptdadecenoic (C17 : 1)	<0.14	<0.50	0.20–0.70	0.03–0.30	0.01–2.40	<0.2
Oleic (C18 : 1n–9)	4.75–26.60	18.30–22.40	27.75–122.49	20.60–48.70	1.54–24.72	0.085–29.00
Eicosenoic (C20 : 1)	0.01–0.02	0.34–0.37	0.02–3.50	0.10–0.60	0.024–0.36	<0.04

**PUFA**						

Linoleic (C18 : 2n–6)	3.69–31.40	7.59–36.27	15.60–71.05	12.90–35.58	0.06–41.39	0.04–24.60
Linolenic (C18 : 3n–3)	0.37–3.60	0.90–2.73	0.30–13.10	0.03–10.50	0.007–1.66	0.44–40.70
∑SFA	36.20–87.62	35.89–47.62	34.90–49.65	2.32–30.21	1.10–46.50	3.20–28.80
∑MUFA	8.49–32.10	30.71–31.40	28.93–42.40	2.51–53.20	0.76–29.99	1.28–27.70
∑PUFA	4.62–58.70	29.14–39.85	15.70–31.80	5.85–48.31	0.45–42.64	3.55–43.60
n-6	13.33–25.80	2.91	16.50–24.00	11.49–22.38	1.05–42.63	1.87–3.13
n-3	1.40–3.20	0.95–13.41	0.78–2.22	0.42–1.86	0.01–2.49	0.63–1.68
n-6/n-3	0.015–6.05	0.22–3.12	0.08–27.38	1.40–38.60	2.00–40.90	1.11–4.97
References	[62, 63, 70, 73, 117, 123, 148, 190–196]	[73, 75, 126, 152, 197]	[71, 81, 84, 80, 82, 85, 129, 153, 154, 198, 199, 123, 131, 200]	[71, 81, 82, 85, 91, 129, 87, 201, 202, 3, 50, 140, 203, 156, 204, 205, 132]	[95, 129, 161, 206–208, 50, 53, 209, 98, 100, 123, 136]	[129, 164, 210–213, 101, 139, 140, 141, 142, 144]

Abbreviations: AD, *Acheta domesticus*; BM, *Bombyx mori*; HI, *Hermetia illucens*; MD, *Musca domestica*; MUFAs, monounsaturated fatty acids; NR, not reported; PUFAs, polyunsaturated fatty acids; SFAs, saturated fatty acids; TM, *Tenebrio molitor*; ZM, *Zophobas morio*.

**Table 5:** Amino acid requirements (g/100 g protein) of different fish species.*⁣*^*∗*^

Nutrient	Rainbow trout (*Oncorhynchus mykiss*)	Atlantic salmon (*Salmo salar*)	Pacific salmon (*Oncorhynchus* sp.)	Common carp (*Cyprinus carpio*)	Nile tilapia (*Oreochromis* sp.)	European sea bass (*Dicentrarchus labrax*)
Valine	1.20	1.20	1.20	1.40	1.50	NR
Isoleucine	1.10	1.10	1.00	1.00	1.00	NR
Leucine	1.50	1.50	1.60	1.40	1.90	NR
Lysine	2.40	2.40	2.20	2.20	1.60	2.20
Threonine	1.10	1.10	1.10	1.50	1.10	1.20
Phenylalanine	0.90	0.90	0.90	1.30	1.10	NR
Methionine	0.70	0.70	0.70	0.70	0.70	NR
Histidine	0.80	0.80	0.70	0.50	1.00	NR
Tryptophan	0.30	0.30	0.30	0.30	0.30	0.30
Arginine	1.50	1.80	2.20	1.70	1.20	1.80

Abbreviation: NR, not reported.

*⁣* ^*∗*^ [254].

**Table 6:** Insect-protein sources as a substitute for FM in the diet of different aquatic species.

Insect species	Fish species	Inclusion level (%)	Duration (weeks)	Main results	Reference
HI	Nile tilapia (*Oreochromis niloticus*)	10 100	12	Performance: Growth and feed efficiency maintenance; immunity: ↑ skin immune response	[313]
HI	Zebrafish (*Danio rerio*)	25 50	24	Performance: ↓ Growth and slight lipid accumulation; health: no negative effects on immunity or intestinal histology.	[314]
HI	Rainbow trout (*Oncorhynchus mykiss*)	15	12	Performance: Growth maintenance and protein content retention.	[123]
HI	Tambaqui (*Colossoma macropomum*)	50 100	18	Performance: Growth similar to control. Quality: ↑ Sensory acceptance of fillets.	[315]
HI	Yellow catfish (*Pelteobagrus fulvidraco*)	10 30	8	Performance: Up to 20% replacement with no negative impact on growth. Health: 30% Inclusion ↑ plasma cholesterol.	[316]
HI	Rainbow trout (*Oncorhynchus mykiss*)	20 40	11	Performance: No adverse effects on growth and digestibility. Nutritional profile: ↓ Polyunsaturated fatty acids.	[116]
HI	Nile tilapia (*Oreochromis niloticus*)	50 100	13	Performance: Growth comparable to control; good feed conversion. Health: Stable hematological parameters.	[317]
HI	Mirror carp (*Cyprinus carpio* *var. specularis*)	0 17.47	8	Performance: No significant changes in growth. Body composition: ↓ Lipid content. Health: ↑ Antioxidant activity.	[318]
HI	Grass carp (*Ctenopharyngodon idellus*)	0 25 50 75 100	8	Health: ↑ Antioxidant capacity. Digestive system: Substitution >50% causes intestinal alterations.	[319]
HI	European seabass (*Dicentrarchus labrax*)	Up to 19.5	9	Performance: No effect on growth and digestibility.	[320]
HI	Atlantic salmon (*Salmo salar*)	100	16	Health: No compromise in intestinal health. Performance: No effect on growth	[321]
MD	African catfish (*Clarias gariepinus*)	0 25 50 75 100	12	Performance: 75% Inclusion ↑ weight gain (50.71 g), ↓ FCR (1.04), and ↑ SGR (1.99 g/day). Economics: ↓ Production cost	[322]
MD	Red seabream (*Pagrus major*)	0.5 5	3	Performance: ↑ Growth. Immunity: ↑ Peritoneal leukocyte phagocytic activity	[323]
MD	Nile tilapia (*Oreochromis niloticus*)	0 25 50 75 100	12	Performance: Higher inclusion levels led to growth similar to control. Quality: No negative effects on meat quality.	[324]
MD	African catfish (*Clarias gariepinus*)	0 25 50 75 100	8	Performance: ↑ Growth with 75% inclusion. Health: No negative effects on liver and kidney histology	[325]
MD	Hybrid catfish (*Clarias gariepinus × Heterobranchus longifilis*)	14 21	6	Performance: ↑ Final weight. Health: ↑ Immunity and antioxidant levels. Feeding behavior: No impact on feed intake.	[326]
MD	Nile tilapia (*Oreochromis niloticus*)	20–80	10	Performance: 50%–60% Inclusion ↑ growth rate and feed conversion. Survival: 100% Survival rate.	[327]
MD	Gibel carp (*Carassius auratus gibelio*)	39	6	Performance: No effect on growth. Health: ↑ Antioxidant capacity in liver and intestine.	[328]
MD	Nile tilapia (*Oreochromis niloticus*)	66	8	Performance: Dehydrated form ↑ growth and weight gain; fresh form slightly ↓ growth. Feed efficiency: No difference in feed conversion ratio.	[329]
MD	African catfish (*Clarias gariepinus*)	0 12.5 50 100	8	Feed efficiency: Best FCR with 25% inclusion. Performance: ↓ Growth with 100% substitution. Protein utilization: Highest efficiency with 50% substitution.	[330]
MD	Nile tilapia (*Oreochromis niloticus*)	35	12	Performance: Larvae grown on *Eucheuma* ↑ growth. Nutritional profile: ↑ Omega-3 fatty acid composition in fish.	[331]
ZM	Rainbow trout (*Oncorhynchus mykiss*)	25	8	Performance: No significant differences in growth, FCR, and gross energy utilization. Nutritional profile: ↓ n-3 Fatty acid content in fish fed with insects.	[100]
ZM	Goldfish (*Carassius auratus*)	25–100	24	Performance: Increasing *Z. morio* inclusion ↑ growth. Digestion: Improved digestibility. Quality: ↑ Carotenoid deposition.	[322]
ZM	Gilthead seabream (*Sparus aurata*)	5–30	14	Immunity: Up to 20% inclusion ↑ immunomodulation. Health: 30% Inclusion ↑ nitric oxide production.	[332]
ZM	Rainbow trout (*Oncorhynchus mykiss*)	15	12	Performance: No effect on growth. Nutritional profile: Higher protein retention rate than other insect-based diets.	[123]
ZM	Cobia (*Rachycentron canadum*)	50	8	Performance: No significant impact on growth. Nutrition: No significant impact on feed conversion, or protein digestibility compared to the control diet.	[333]
ZM	Rainbow trout (*Oncorhynchus mykiss*)	25	6	Performance: ↑ Growth and feed conversion rate. Health: No adverse effects on liver, kidney, and intestinal histology.	[334]
ZM	Guppy (*Poecilia reticulat*a)	100	12	Performance: No effect on growth Survival: No effect on survival rate compared to commercial feed.	[335]
ZM	Red tilapia (*Oreochromis spp*.)	30	8	Digestibility: ↓ Protein and lipid digestibility compared to the FM control group. Feed efficiency: Lower than fishmeal.	[131]
ZM	Gilthead seabream (*Sparus aurata*)	10–30	12	Performance: No negative impact on growth. Feed efficiency: ↑ Feed conversion. Immunity: ↑ Immune response.	[336]
TM	European seabass (*Dicentrarchus labrax*)	40 80 100	10	Performance: No impact on feed intake and weight gain. Health: ↓ Plasma cholesterol at 40% substitution.	[337]
TM	Grass carp (*Ctenopharyngodon idellus*)	25 50 75 100	8	Performance: 25% Substitution ↑ growth and protein efficiency. Health: Total replacement caused oxidative stress and liver dysfunction.	[338]
TM	Apistogramma (*Apistogramma agassizii*)	50 100	6	Performance: No significant differences in the evaluated zootechnical parameters.	[339]
TM	European seabass (*Dicentrarchus labrax*)	50 100	10	Feedingbehavior: No change in feed intake in the short and medium term. Health: ↑ Liver triglyceride levels in the long term.	[340]
TM	African catfish (*Clarias gariepinus*)	20 40 60 80 100	7	Performance: Up to 40% substitution had no impact on growth. Feed efficiency: 80% Maintained good growth and feed efficiency. Growth: 100% Substitution slightly reduced growth.	[341]
TM	Mandarin fish (*Siniperca scherzeri*)	20 30	8	Performance: ↑ Growth at 20% inclusion. Feed efficiency: ↓ At 30% inclusion.	[342]
TM	Rainbow trout (*Oncorhynchus mykiss*)	25 50	8	Quality: No impact on morphological and commercial traits. Nutritional profile: ↓ EPA and DHA; ↑ saturated fatty acids.	[343]
TM	Sea trout (*Salmo trutta m. trutta*)	20	8	Performance: No effect on growth Digestion: No effect on gastrointestinal enzyme activity.	[344]
TM	Large yellow croaker (*Larimichthys crocea*)	15 30	8	Performance: 15% Inclusion had no impact on growth; 30% ↓ growth. Immunity: 15% Inclusion ↑ intestinal immunity.	[345]
TM	Rainbow trout (*Oncorhynchus mykiss*)	25 50	12	Digestibility: No effect on protein digestibility Composition: No effect on muscle composition. Performance: 50% Inclusion ↓ growth.	[346]
AD	Nile tilapia (*Oreochromis niloticus*)	100	6	Performance: ↓ Weight gain and feed conversion rate compared to the control diet. Survival: No effect on survival rate	[347]
AD	Nile tilapia (*Oreochromis niloticus*)	25 35	6	Performance: Diet with 35% house cricket showed similar performance to commercial feed..	[348]
AD	Red hybrid tilapia (*Oreochromis spp*.)	60 70 80 90 100	4	Performance and survival: Best growth and survival rate with 60% *A. domesticus* + 40% rice bran. Health: >70% Inclusion ↓ growth and caused liver alterations.	[349]
AD	Green swordtail (*Xiphophorus helleri*)	100	10	Performance: Growth similar to control. Health: Liver alterations observed.	[350]
BM	Nile tilapia (*Oreochromis niloticus*)	0 33.3 66.6 100	14	Performance: Up to 66.6% substitution had no effect on growth, feed conversion, or body composition. Efficiency: 100% Substitution ↓ performance and protein efficiency	[351]
BM	African catfish (*Clarias gariepinus*)	0 25 50 75 100	6	Growth: Up to 50% substitution ↑ growth and feed efficiency. Performance: 100% Substitution resulted in lower performance than control.	[352]
BM	African catfish (*Clarias gariepinus*)	25 50 75 100	4	Best growth and feed efficiency at 50% inclusion. 100% Substitution ↓ growth and efficiency.	[353]
BM	Gift tilapia (*Oreochromis niloticus*)	10 20 40	10	40% Inclusion ↑ WG, FER, organosomatic indices, and blood parameters. No impact on crude protein.	[354]
BM	Walking catfish (*Clarias batrachus*)	25 50 75 100	8	Growth and feed utilization in 100% BM group similar to fish meal diet. 100% BM was the most cost-effective.	[355]
BM	Mirror carp (*Cyprinus carpio var. specularis*)	4 8 12 16	8	Up to 40 g/kg BM had no adverse effects. ≥80 g/kg ↓ growth, feed utilization, and lipid metabolism.	[356]
BM	Rainbow trout (*Oncorhynchus mykiss*)	5 10 15 20	8	Performance: Up to 10% substitution had no effect on growth, feed conversion, or body composition. Efficiency: 15% Substitution ↓ feed efficiency and growth rate.	[357]

*Note*: ↑, Increase/improvement; ↓, decrease/reduction; n-3, omega-3 fatty acids.

Abbreviations: AD, *Acheta domesticus*; BM, *Bombyx mori*; CL, crude lipid; CP, crude protein; DHA, docosahexaenoic acid; EPA, eicosapentaenoic acid; FCR, feed conversion ratio; FER, feed efficiency ratio; HI, *Hermetia illucens*; MD, *Musca domestica*; PER, protein efficiency ratio; SGR, specific growth rate; SR, survival rate; TM, *Tenebrio molitor*; WG, weight gain; ZM, *Zophobas morio*.

## Data Availability

Data sharing is not applicable to this article as no new data were created or analyzed in this study.
